# Editorial Comment: Laparoscopy versus robotic-assisted pyeloplasty in children: preliminary results of a pilot prospective randomized controlled trial

**DOI:** 10.1590/S1677-5538.IBJU.2020.04.05

**Published:** 2020-05-20

**Authors:** Eliney F. Faria

**Affiliations:** 1 Serviço de Urologia Hospital Felicio Rocho Belo HorizonteMG Brasil Serviço de Urologia, Hospital Felicio Rocho, Belo Horizonte, MG, Brasil

Silay MS ^1,2^, Danacioglu O ^3^, Ozel K ^4^, Karaman MI ^3^, Caskurlu T ^3^

^1^ Department of Pediatric Urology, Istanbul Gelisim University & Istanbul Memorial Hospital, Istanbul, Turkey; ^2^ Istanbul Bahcelievler Memorial Hastanesi, Bahcelievler, Istanbul, Turkey; ^3^ Department of Urology, Istanbul Medeniyet University, Istanbul, Turkey; ^4^Department of Pediatric Surgery, Istanbul Medeniyet University, Istanbul, Turkey

World J Urol. 2019 Aug 22. [Epub ahead of print]

**DOI: 10.1007/s00345-019-02910-8 | ACCESS: 10.1007/s00345-019-02910-8**

## COMMENT

This interesting paper reported a prospective randomized controlled trial (RCT) about laparoscopic and robotic pyeloplasty in the treatment of ureteropelvic junction obstruction (UPJO) in children. They addressed if the robotic-assisted laparoscopic pyeloplasty (RALP) has additional advantages over conventional laparoscopic pyeloplasty (LP) regarding suturing, comfort for the surgeon and visualization. The main disadvantage of RALP is its higher cost ( 1 , 2 ). This is the first RCT comparing LP and RALP in pediatric population. In a period of 2 years, a total of 53 children (0–18 years old) with UPJO were enrolled into the RCT for either LP or RALP (Group 1, n: 27 - Group 2, n:26). The presence of crossing vessel was identified in 7 (25.9%) patients for LP group and in 6 (23.1%) patients for RALP group. Mean total operative time in LP group was 139.26 ± 43.21 min (80–250 min) compared to 105.19 ± 22.87 min (70–150 min) in RALP group (p = 0.001). The number of the trocar placement was significantly less in LP group (mean 3.00 ± 0) compared to RALP group (mean 3.81 ± 0.40) (p = 0.001). The mean cost of RALP was higher than LP (p = 0.001). They completed successfully all cases with none converted to open surgery. Postoperative complication rates were similar for both groups in the follow-up period. They reported overall success rate of 96.2%, similar to previously published series of minimally invasive pyeloplasty. Accordingly, robotic procedures had approximately four times higher cost than conventional laparoscopy ( 3 ). Despite small number of patients there was a as a pilot study, they reported a RCT and their findings are important to demonstrate the comparison of LP and RALP in children. The short-term results reveals that both LP and RALP are safe and effective in children with comparable success and complication rates.
